# Oncolytic Vaccinia Virus Armed with GM-CSF and IL-7 Enhances Antitumor Immunity in Pancreatic Cancer

**DOI:** 10.3390/biomedicines13040882

**Published:** 2025-04-05

**Authors:** Wenyi Yan, Yujing Xuan, Ruimin Wang, Ziyan Huan, Yu Guo, Huilin Dun, Lihua Xu, Ruxia Han, Xianlei Sun, Lingling Si, Nicholas Robert Lemoine, Yaohe Wang, Pengju Wang

**Affiliations:** 1 Sino-British Research Centre for Molecular Oncology, National Centre for International Research in Cell and Gene Therapy, State Key Laboratory of Esophageal Cancer Prevention & Treatment, School of Basic Medical Sciences, Academy of Medical Sciences, Zhengzhou University, Zhengzhou 450001, China; ywy201377@126.com (W.Y.); yujing_xuan@126.com (Y.X.); hziyan396@163.com (Z.H.); guoyu0320@163.com (Y.G.); dunhuilin2022@163.com (H.D.); xulihuayky@zzu.edu.cn (L.X.); hanahrx@zzu.edu.cn (R.H.); sunxianlei@zzu.edu.cn (X.S.); silingling@zzu.edu.cn (L.S.); nick.lemoine@nihr.ac.uk (N.R.L.); yaohe.wang@qmul.ac.uk (Y.W.); 2 Department of Pathology, Zhengzhou People’s Hospital, Fifth Clinical Medical College of Henan University of Chinese Medicine, Zhengzhou 450003, China; 202012402015866@gs.zzu.edu.cn; 3 Centre for Biomarkers & Biotherapeutics, Barts Cancer Institute, Queen Mary University of London, London EC1M 6BQ, UK

**Keywords:** oncolytic virus, vaccinia virus, GM-CSF, IL-7, pancreatic cancer

## Abstract

**Objectives:** Pancreatic cancer remains a therapeutic challenge due to its immunosuppressive microenvironment and treatment resistance. This study aimed to develop a novel recombinant oncolytic vaccinia virus (VVL-GL7) co-expressing granulocyte-macrophage colony-stimulating factor (GM-CSF) and interleukin-7 (IL-7), designed to enhance anti-tumor immunity and synergize with immune checkpoint inhibitors. **Methods:** VVL-GL7 was constructed through CRISPR/Cas9-mediated knockout of TK and A49 genes, combined with the simultaneous insertion of dual cytokine-encoding cassettes. Anti-tumor efficacy was evaluated in vitro and in vivo using C57BL/6 mouse and Syrian hamster pancreatic cancer models. Comprehensive immune profiling evaluated CD8^+^ T-cell and macrophage infiltration dynamics while simultaneously assessing memory T-cell differentiation patterns using flow cytometry. Preclinical combination studies of VVL-GL7 and the PD-1 immune checkpoint inhibitor were systematically evaluated in a syngeneic pancreatic cancer model. **Results:** VVL-GL7 exhibited potent oncolytic activity, inducing significant tumor regression in both preclinical models. VVL-GL7 therapy significantly augmented CD8^+^ T-cell and macrophage infiltration within the tumor microenvironment, while concomitantly driving memory T-cell differentiation. The synergistic effects of VVL-GL7 and the PD-1 blockade further improved therapeutic outcomes, resulting in significantly higher tumor remission rates compared to monotherapy and achieving complete tumor regression in pancreatic cancer models. **Conclusions:** VVL-GL7 reprograms the immunosuppressive tumor microenvironment and synergizes with anti-PD-1 antibodies to overcome resistance in pancreatic cancer.

## 1. Introduction

Across the globe, cancer ranks among the primary factors contributing to mortality. The current mainstay therapies, including chemotherapy and radiotherapy, although effective, are often accompanied by severe side effects and have diminished therapeutic returns in advanced or recurrent tumors [1]. Cancer immunotherapy has made marked progress in recent years, especially the successful use of immune checkpoint inhibitors (ICIs, e.g., PD-1 and CTLA-4 antibodies) in many types of tumors [2]. While these treatment modalities have demonstrated remarkable clinical successes, a significant subset of individuals fail to exhibit therapeutic responses, highlighting persistent limitations in current approaches. Pancreatic adenocarcinoma, characterized by the most dismal prognosis and lowest survival rates among all malignancies, exhibits therapeutic resistance to immune checkpoint inhibitor (ICI) therapy due to its highly immunosuppressive tumor microenvironment (TME) coupled with stroma-rich physical barriers that impede immune cell infiltration [1,3,4]. In recent years, the oncolytic virus (OV) has gained much attention as an emerging strategy for tumor therapy [5,6]. OVs constitute a class of bioengineered or naturally occurring viral agents that exhibit preferential tropism for neoplastic cells. Through targeted cellular lysis, these therapeutic vectors facilitate the release of tumor-associated antigens, thereby initiating cascades of host-derived antitumor immunity [7,8]. T-VEC, a first-in-class recombinant herpes simplex virus (HSV) engineered to express granulocyte-macrophage colony-stimulating factor (GM-CSF), achieved landmark therapeutic certification in 2015 as the pioneering oncolytic viral agent granted clinical authorization by regulatory agencies [9]. This milestone significantly advanced the clinical acceptance of oncolytic viruses (OVs) as legitimate therapeutic agents. Multiple oncolytic viral platforms, including vaccinia virus, adenovirus, and herpes simplex virus, have undergone clinical trials and shown anti-tumor efficacy to some extent [10,11,12].

Vaccinia virus (VV) is an extensively researched oncolytic virus that, after genetic modifications, has demonstrated significant potential as an oncolytic viral vector, primarily due to its large genome-carrying capacity and the ability to be engineered for enhanced tumor selectivity [13]. The thymidine kinase (TK, J2R) gene in VV is intimately associated with viral replication. Its elimination markedly curtails viral replication in normal cells, thereby enhancing the selectivity of the virus in tumor cells [14,15,16]. Rational genetic modifications, such as the removal of certain immunosuppressive genes, can further enhance its antitumor efficacy [17,18]. The A49 gene, which suppresses the antiviral immune response by inhibiting the NF-κB pathway, is one of the key immunomodulatory genes in VV. Deletion of the A49 gene can alleviate this suppression, thereby reducing viral virulence [19]. Our investigations revealed that VVL-DD (a Lister strain vaccinia virus engineered with dual TK and A49 gene deletions) demonstrated enhanced tumor-targeting specificity in cellular assays and superior therapeutic efficacy in animal models relative to the single-gene modified VVLΔTK construct during evaluations in pancreatic adenocarcinoma murine systems [20].

Although the deletion of endogenous genes improved the antitumor ability of OV, relying on the antitumor immune response stimulated by the virus itself alone may not be sufficient to achieve a long-lasting antitumor effect [21]. Therefore, enhancing the immune activity of viral vectors through the introduction of exogenous immunomodulatory factors has become a feasible strategy [22]. GM-CSF enhances antigen presentation ability by promoting the growth and functional maturation of dendritic cells and macrophages, thereby inducing T-cell activation and clonal proliferation within the tumor microenvironment [23,24,25]. GM-CSF has been widely used to enhance anti-tumor immune responses of OVs, such as T-VEC, JX594 (VV), and ONCOS-102 (adenovirus) and proved safe in clinical trials [26,27,28,29]. Clinical evaluation of the thymidine kinase-deficient Wyeth strain vaccinia vector (JX-594/Pexa-Vec) engineered to co-express GM-CSF demonstrated tumor-selective replication patterns in phase II sarcoma trials. Although intralesional and intravenous administrations showed acceptable safety profiles, the observed therapeutic responses failed to reach predefined clinical endpoints [29,30]. Interleukin-7 (IL-7), a critical cytokine in adaptive immunity, has been extensively utilized to amplify tumor-targeting T-cell functionality, with pronounced effects on CD8^+^ cytotoxic T lymphocyte activation and persistence [31,32,33]. IL-7 is crucial in the establishment and maintenance of immune memory, and thus its introduction into oncolytic viruses can be expected to significantly enhance the durability of the immune response to tumors [34,35,36,37].

In this study, we first investigated the antitumor mechanism of VVL-DD in a murine pancreatic cancer model. Based on the changes in the TME after VVL-DD treatment, we inserted GM-CSF and IL-7 into this backbone virus to create VVΔTK-GM-CSFΔA49-IL-7 (hereafter, VVL-GL7) and systematically evaluated the anti-tumor efficacy of the recombinant vaccinia viruses in murine pancreatic cancer model in vivo and in vitro. In addition, to investigate the effects of different viral treatments on the TME and systemic immune cells, we used flow cytometry and immunohistochemistry to comprehensively analyze the composition of immune cells in tumor tissues, spleens, lymph nodes, and peripheral blood, especially the dynamics of memory T-cells and myeloid immune cells. Our experimental data demonstrate that the simultaneous delivery of GM-CSF and IL-7 potentiates vaccinia virus (VV)-mediated tumor regression through increased infiltration and functional activation of tumor-associated immune cell populations.

## 2. Materials and Methods

### 2.1. Cell Lines

In this study, we used CV1 (African green monkey kidney cells); murine pancreatic ductal adenocarcinoma (PDAC) cell lines DT6606, DT4994 (both from *Kras*^LSL-G12D/+^; *T*Pdx1-Cre KPC mice), and TB11381 (from *Kras*^LSL-G12D/+^; *Trp53*^LSL-R172H/+^; Pdx1-Cre KPC mice); Syrian hamster pancreatic cancer cell lines SHPC6, HPD1NR, and Hap-T1; human lung adenocarcinoma cell line A549; human colon cancer cell line HCT116; human pancreatic cancer cell lines ASPC1 and 8988T; and human embryonic lung fibroblast cell line MRC-5. All these cell lines were provided and preserved by the International Joint Research Center for Cell and Gene Therapy, Zhengzhou University, Zhengzhou, China.

CV1, MRC-5, DT6606, TB11381, DT4994, HCT116, A549, ASPC1, 8988T, SHPC6, and Hap-T1 were cultured in DMEM medium (Gibco, Grand Island, NE, USA), while HPD1NR was cultured in RPMI-1640 medium (Gibco, Grand Island, NE, USA). Each medium was supplemented with 10% fetal bovine serum (FBS) (PAN-Biotech GmbH, Aidenbach, Germany), 1% streptomycin, and 1% penicillin (Solarbio, Beijing, China). All cell lines were cultured under standard conditions (37 °C, 5% CO_2_, humidified atmosphere) and preserved in a cryogenic storage medium containing 90% fetal bovine serum (FBS) supplemented with 10% dimethyl sulfoxide (DMSO) (Solarbio, Beijing, China).

### 2.2. Viruses

Vaccinia virus Lister (VV-L) served as the genomic backbone for targeted engineering through CRISPR/Cas9-mediated homologous recombination [8,20]. VVLΔTK, VVL-DD, and VVLΔTK-GM-CSFΔA49 (hereafter referred to as VVL-GF) were provided by the International Joint Research Center for Cell and Gene Therapy, Zhengzhou University. The shuttle vector pUC-A49 and plasmid pUC-IL-7 (Azenta Life Sciences, Suzhou, China) were commercially sourced from GENEWIZ (Suzhou, China) and pre-engineered with Sal I/Nhe I restriction sites for viral vector construction. Following enzymatic digestion of pUC-IL-7 with Sal I and Nhe I, the IL-7 coding sequence was directionally ligated into the corresponding restriction sites of the pUC-A49 shuttle backbone. Homologous recombination was employed to generate the following recombinant viruses: VVLΔTKΔA49-IL-7 and VVLΔTK-GM-CSFΔA49-IL-7 (hereafter referred to as VVL-IL7 and VVL-GL7, respectively). Depending on the species origin of the inserted exogenous genes, these viruses were classified as human-origin vaccinia viruses (VVLΔTK-hGM-CSFΔA49R-hIL-7 and VVL-hGL7) or mouse-origin vaccinia viruses (VVLΔTK-mGM-CSFΔA49R-mIL-7 and VVL-mGL7), along with their respective control viruses.

### 2.3. Viral Replication Assay and Cytotoxicity Assay

Tumor cells were digested, counted, and suspended in DMEM or the 1640 medium containing 2% FBS. The cells were seeded at 2 × 10^5^ cells in 2 mL per well in 12 wells of two 6-well plates. Then, 14–18 h later, the required viral load was calculated based on a multiplicity of infection (MOI) of 1 pfu/cell. The virus was then diluted accordingly and directly added to the wells. At 24, 48, and 72 h post-infection, the cells and supernatant were collected together and stored at −80 °C, with triplicate wells prepared for each time point. The viral load in these cell lysates was later quantified through the 50% tissue culture infectious dose (TCID50) assay. Each sample from the initial infection was tested in triplicate for each time point. After incubating for 14–18 h, the cell lysates collected earlier were retrieved from the −80 °C freezer, thawed in a 37 °C water bath, and subjected to three cycles of freeze–thawing using liquid nitrogen and a 37 °C water bath to lyse the cells and release the virus.

In the reinfection assay, CV1 cells were prepared in 10% DMEM, and 5000 cells in 180 µL per well were seeded into a 96-well plate. The viral titer in these lysates was then determined by serially diluting the samples from 1:10 to 1:10^6^ and using the TCID50 method.

To determine viral-mediated cytotoxic effects on cellular models, triplicate measurements were processed for viability analysis. Following a 6-day incubation, post-viral infection cell viability was quantified using an MTS assay kit (Promega, Madison, WI, USA, Cat# G3580), with experimental procedures strictly adhering to the supplier’s protocol.

### 2.4. Flow Cytometry Analysis

Tumor specimens underwent mechanical dissociation followed by enzymatic processing. Minced tissue fragments were subjected to dual-enzyme digestion (Collagenase IV/I cocktail, 2 mg/mL total concentration) (Sigma-Aldrich, St. Louis, MO, USA) in RPMI-1640 at 37 °C with shaking (120 rpm, 40 min). Post-digestion suspensions were centrifuged (300× *g*, 5 min) and sequentially passed through 70 μm nylon mesh. Cell viability exceeded 85% (trypan blue exclusion assay) prior to subsequent analyses. Spleen tissue and inguinal lymph nodes were smashed and washed twice with PBS in a 6-well plate and ground into a cell suspension. The suspension was filtered through a 70 μm filter and centrifuged at 2000 rpm for 5 min at 4 °C. After discarding the supernatant, splenocytes were resuspended in 1 mL of erythrocyte lysing buffer (1×) per tube (Solarbio, Beijing, China), while lymph node cells were resuspended in 1 mL of PBS per tube and kept on ice. After incubation, the suspensions were centrifuged again at 2000 rpm for 5 min at 4 °C, the supernatant was discarded, and the cells were washed twice with PBS. Finally, cells were resuspended in 1 mL of 2% staining buffer (PBS with 2% FBS), filtered through a 70 μm filter, and transferred to new 1.5 mL tubes. Cells were counted, aliquoted (1 × 10^6^ cells/tube), and centrifuged again.

Single-cell suspensions were prepared for multiparametric immunophenotyping. To reduce nonspecific antibody interactions, cells were pre-incubated with an Fc-blocking reagent (TruStain FcX™) for 10 min at 4 °C. Surface antigen staining was performed using the following fluorochrome-conjugated antibody panel:

T-cell subsets: CD3ε (FITC, clone 17A2), CD4 (BV510™, clone GK1.5), CD8α (BV650™, clone 53-6.7).

Activation markers: CD25 (APC, clone PC61), CD127 (BV605™, clone A7R34), CD122 (PE/Cy7, clone TM-β1).

Myeloid populations: CD11b (FITC, clone M1/70), CD86 (BV785™, clone GL1), F4/80 (PE/Cy7, clone BM8).

Antigen-presenting cells: MHC II (APC, clone M5/114.15.2), CD11c (BV421™, clone N418).

Cell suspensions were co-stained with Zombie NIR™ Fixable Viability Dye (1:1000) in PBS-based staining buffer for 30 min at 4 °C under light-protected conditions. All antibodies were sourced from BioLegend (San Diego, CA, USA) and titrated according to the manufacturer’s recommendations. Following two washes with PBS containing 0.5% BSA, cells were resuspended in PBS for immediate acquisition. Fluorescence compensation and data analysis were conducted using FlowJo v10.8 (FlowJo LLC, Ashland, OR, USA).

### 2.5. Animal Studies

All experimental protocols involving vertebrate animals were authorized under ethical approval code V3A02022000001 by the Zhengzhou Laboratory Animal Governance Center. C57BL/6J mice and golden Syrian hamsters were purchased from Beijing Charles river Co., Ltd. (Beijing, China). They were housed in IVC cages, maintained on a standard diet, and were provided water ad libitum throughout the experiments under specific pathogen-free conditions.

For therapeutic evaluation, 6-week-old C57BL/6 hosts received dorsal flank implants of 2 × 10^6^ TB11381/DT6606 cells. Upon attaining target volumes (80–120 mm^3^), subjects received intralesional administrations of either PBS or viral suspensions 3 times every other day. In combination cohorts, the PD-1 blockade (clone 29F.1A12, 200 μg/dose) was delivered intraperitoneally twice a week alongside oncolytic virotherapy (VVL-GL7, 10^8^ PFU/dose on days 0/2/4).

Syrian hamster peritoneal carcinomatosis models were established via intraperitoneal inoculation of 10⁷ SHPC6 cells [38]. Therapeutic agents (PBS or 10^7^ PFU recombinant vectors expressing humanized GM-CSF/IL-7) were administered on days 0/2/4 post-implantation.

Subcutaneous neoplastic growth was quantified via vernier caliper measurements, with volumetric estimations derived from the modified ellipsoid formula V = (L × W^2^)/2, where L = long-axis diameter, W = orthogonal short-axis diameter. Health-monitoring protocols mandated euthanasia upon reaching terminal endpoints: cachectic manifestations, ≥20% body mass reduction, or lesion volume exceeding 2 cm^3^. Complete response (CR) was defined as the absence of palpable masses.

### 2.6. ELISA

Murine PDAC cell lines (DT6606 and TB11381) were plated in three technical replicates. Following 16 h incubation for adherence, a viral challenge was initiated at a multiplicity of infection (MOI) of 1 plaque-forming unit (PFU) per cell. Conditioned media were harvested at sequential timepoints (24/48/72 h), with secreted cytokine profiles quantified via the Mouse GM-CSF ELISA Kit (Invitrogen, Carlsbad, CA, USA) and the Mouse IL-7 ELISA kit (Multi Sciences, Hangzhou, China).

### 2.7. Immunohistochemistry and H&E

Tumor specimens were initially fixed in 4% paraformaldehyde (PFA, Sigma-Aldrich, St. Louis, MO, USA) for 24 h at 4 °C, followed by sequential dehydration in graded ethanol solutions (70% to 100%) and paraffin embedding. Tissue blocks were sectioned into 3.5 μm slices with microtome (Leica RM2245, Leica Biosystems, Wetzlar, Germany).

For hematoxylin and eosin (H&E) staining, slides were first deparaffinized in xylene (three cycles of 5 min each) and rehydrated through a descending ethanol series (100% to 70%). Nuclear staining was performed by immersing sections in Mayer’s hematoxylin (Thermo Fisher Scientific, Waltham, MA, USA) for 8 min, followed by differentiation in 0.1% HCl-ethanol. Cytoplasmic counterstaining was achieved using eosin Y (Thermo Fisher Scientific, Waltham, MA, USA) (0.5% in 95% ethanol) for 2 min. Finally, slides were dehydrated in ascending ethanol concentrations (95% to 100%), cleared in xylene, and coverslipped with EcoMount™ (Biocare Medical, Concord, MA, USA).

In immunohistochemical procedures, antigen retrieval was conducted by heating sections in Tris-EDTA buffer (pH 9.0) at 95 °C for 20 min. After protein blocking with 3% BSA/0.05% Tween-20, primary antibodies (1:200) were applied at 4 °C for 16 h. After washing, horseradish peroxidase (HRP)-conjugated secondary antibodies (EnVision™, Agilent, Santa Clara, CA, USA) were incubated for 30 min at room temperature. Chromogenic detection utilized 3,3′-diaminobenzidine (DAB, Dako, Glostrup, Denmark) with reaction termination at an optical density (OD450). Finally, sections were dehydrated through a graded ethanol series, cleared in xylene, and mounted with a permanent medium. Brightfield microscopy images were captured using an Olympus BX53 system and digitally optimized using ImageJ 1.52v software (National Institutes of Health, Bethesda, MD, USA).

### 2.8. Statistical Analysis

All statistical analyses were carried out in GraphPad Prism 9.0 (San Diego, CA, USA). Numerical results are presented as mean ± SEM. Intergroup differences were statistically evaluated using an unpaired Student’s *t*-test (two-group comparisons), a one-way ANOVA (multiple groups), or a two-way ANOVA (factorial designs). Survival probabilities were plotted via the Kaplan–Meier methodology, with between-group significance determined by a log-rank test. A threshold of *p* < 0.05 was applied to define statistical significance across all tests [39].

## 3. Results

### 3.1. VVL-DD Exhibits Enhanced Anti-Tumor Efficacy In Vivo

**Figure 1:**
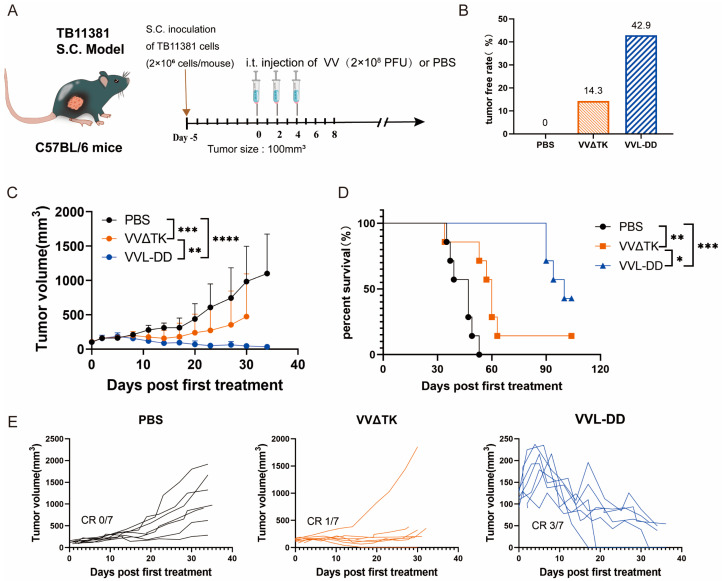
Anti-tumor efficacy of VVL-DD on mouse TB11381 pancreatic cancer model. (**A**) Schematic representation of the treatment protocol. C57BL/6 mice were injected s.c. with 2 × 10^6^ TB11381 cells. When tumor volume reached 100 mm^3^, the mice were randomly assigned to three groups (n = 7). The mice were injected intratumorally with 2 × 10^8^ PFU VVΔTK or VVL-DD or an equivalent volume of PBS every other day; altogether, 5 injections were performed; (**B**) tumor-free rate; (**C**) tumor growth curve. Data are presented as mean ± SD. Statistical significance was calculated using two-way ANOVA. ** *p* < 0.01, *** *p* < 0.001, **** *p* < 0.0001; (**D**) Survival curves. Data were assessed through Kaplan–Meier survival analysis along with log-rank (Mantel-Cox) tests. * *p* < 0.05, ** *p* < 0.01, *** *p* < 0.001; (**E**) Individual tumor growth curve of mice.

Building upon previous work demonstrating the enhanced efficacy of VVL-DD (ΔTK/ΔA49) in murine pancreatic DT6606 tumors (Kras mutation) compared to VVLΔTK [20]. To further evaluate the efficacy of VVL-DD on pancreatic cancer with Kras and p53 mutations, the TB11381 tumor model was established (Figure 1A).

Consistent with prior findings in Kras-mutant DT6606 tumor models, VVL-DD achieved complete tumor regression in three out of seven mice and significantly prolonged survival compared to VVLΔTK (Figure 1C–E). Notably, this study extends the applicability of VVL-DD to tumors with dual Kras/p53 mutations, highlighting its broader utility in genetically heterogeneous pancreatic cancers.

### 3.2. VVL-DD Increases the Accumulation of Macrophages and CD8^+^ T-Cells in the TME

**Figure 2:**
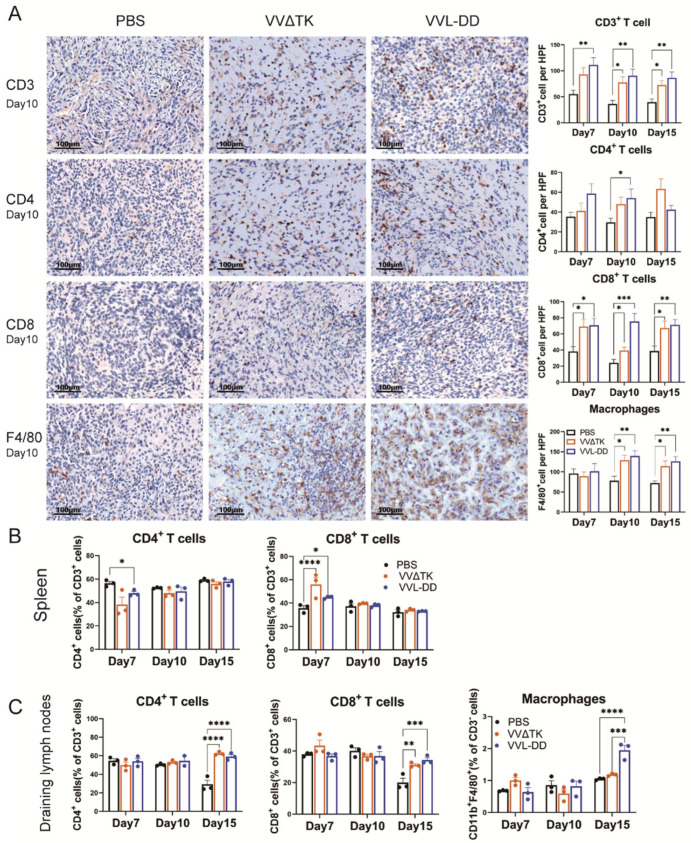
Immune cell profiles of tumor tissue, spleen, and draining lymph nodes of mice after treatments. (**A**) Quantification of immune cell populations in tumor tissues over time post-treatment. Immunohistochemistry (IHC) assay was used to assess the presence of CD3^+^ T-cells, CD4^+^ T-cells, CD8^+^ T-cells, and macrophages (F4/80^+^) on days 7, 10, and 15 post-treatments with PBS, VVΔTK, or VVL-DD. Representative images of IHC staining for CD3^+^ T-cells, CD4^+^ T-cells, CD8^+^ T-cells, and macrophages on day 10. Data are presented as mean ± SEM. * *p* < 0.05, ** *p* < 0.01, *** *p* < 0.001. (**B**,**C**) Spleen and lymph node specimens harvested on post-treatment days 7/10/15; flow cytometric analysis of tissue-derived single-cell suspensions for lymphocyte profiling (n = 3). Data represent mean ± SEM, with two-way ANOVA used for statistical comparisons (* *p* < 0.05, ** *p* < 0.01, *** *p* < 0.001, **** *p* < 0.0001).

To elucidate and improve the therapeutic effects of VVL-DD, we detected immune cell population infiltration into DT6606 subcutaneous tumors. Following the intratumoral injection of VVΔTK and VVL-DD, distinct immune responses were observed in both the tumor microenvironment and systemic immune organs, including the spleen and tumor-draining lymph nodes (Figure 2). We observed a significant increase in CD8^+^ T-cells in the TME of both viral treatment groups compared to the PBS group at all time points, with the VVL-DD group exhibiting the highest levels. This increase in CD8^+^ T-cell infiltration was accompanied by a modest increase in CD4^+^ T-cells, although the latter was less pronounced. CD3^+^ T-cells also gradually increased in both viral groups across all time points, with significant differences compared to the PBS group. In addition to the T-cell response, macrophages (F4/80^+^) showed a significant increase in the VVL-DD group at days 10 and 15, indicating enhanced myeloid cell recruitment (Figure 2A). NK cells, however, remained relatively unchanged across all groups in the tumor microenvironment (Appendix A Figure A2A).

Parallel analysis of the spleen demonstrated a more transient immune activation (Figure 2B). While CD3^+^ T-cell levels remained consistent across all groups, the VVΔTK and VVL-DD groups showed notable increases in CD8^+^ T-cells on day 7, though this effect was not sustained at later time points. In contrast, the CD4^+^ T-cells were reduced in the VVL-DD group on day 7 (Figure 2B). NK cells and macrophages in the spleen also did not show significant changes in any of the treatment groups (Appendix A Figure A2B).

In the tumor-draining lymph nodes, however, both VVΔTK and VVL-DD induced significant immune activation on day 15. Both viral groups showed a significant rise in CD4^+^ and CD8^+^ T-cells compared to the PBS group, suggesting a delayed but robust adaptive immune response in these lymphoid tissues. Furthermore, macrophages also significantly accumulated in the lymph nodes on day 15, particularly in the VVL-DD group, further supporting the notion that this viral vector enhances myeloid cell recruitment (Figure 2C).

Taken together, these findings suggest that VVL-DD induces a stronger and more sustained immune response, particularly in the tumor microenvironment and lymph nodes, with significant recruitment of CD8^+^ T-cells and macrophages, critical for antitumor immunity. The systemic immune response, as observed in the spleen, appears to be more transient and less pronounced, particularly for VVL-DD, highlighting the localized effects of the viral therapy in modulating immune responses in the tumor and draining lymphoid tissues.

### 3.3. Novel Recombinant Vaccinia Virus Expressing GM-CSF and IL-7 Have Better Killing Ability Against Tumor Cells

**Figure 3:**
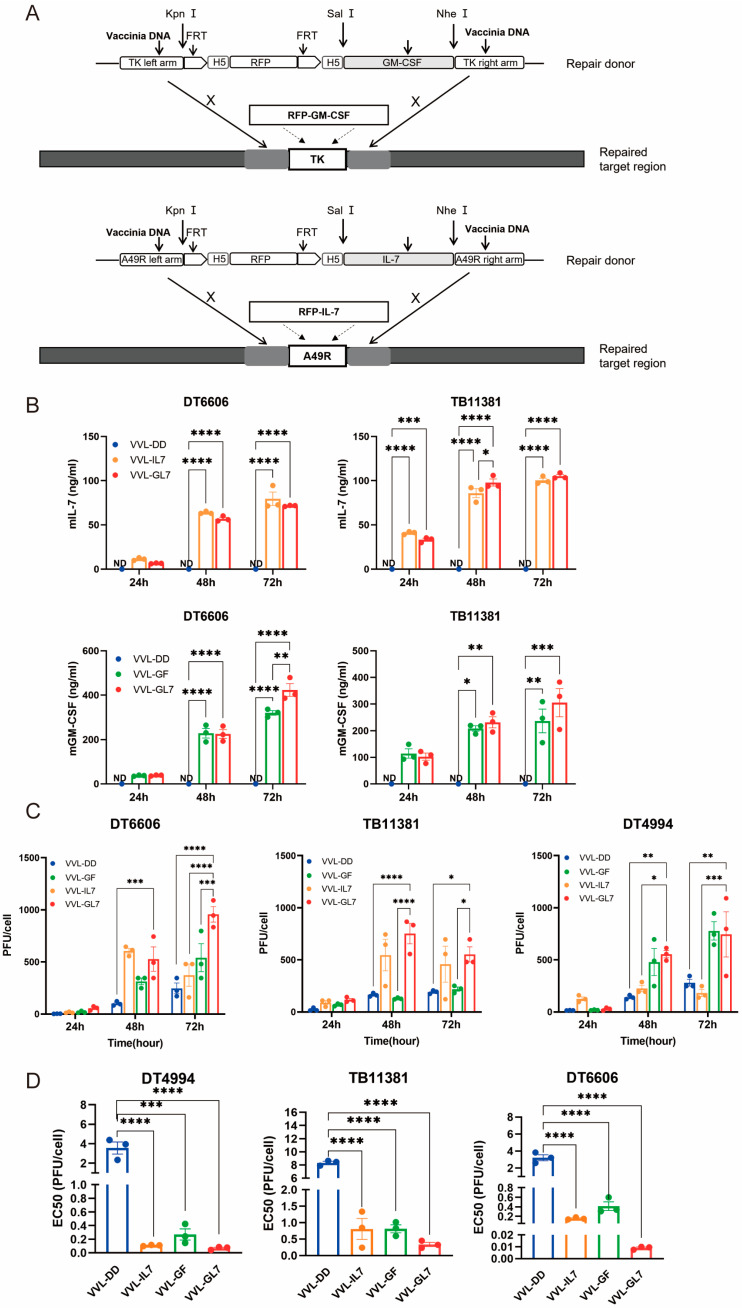
Cytotoxic and replication competency of VVL-GL7 in mouse pancreatic cancer cells. (**A**) Schematic detailing the construction of mutant viruses; (**B**) secreted murine GM-CSF and IL-7 levels in cell culture media were quantified via ELISA at 24, 48, and 72 h following infection with VVL-DD, VVL-IL7, VVL-GF, or VVL-GL7 (MOI = 1 PFU/cell) (n = 3). Data are presented as mean ± SEM. Two-way ANOVA was used for statistical analysis (* *p* < 0.05, ** *p* < 0.01, *** *p* < 0.001, **** *p* < 0.0001). ND, not detected; (**C**) TCID50 quantification in DT6606/TB11381/DT4994 cells (24, 48, and 72 h) (n = 3). Data are presented as mean ± SEM. Two-way ANOVA was used for statistical analysis (* *p* < 0.05, ** *p* < 0.01, *** *p* < 0.001, **** *p* < 0.0001); (**D**) EC50 values of VVL-DD, VVL-GF, VVL-IL7, and VVL-GL7 in DT6606, TB11381, and DT4994 cells (MTS assay; n = 3). Results are reported as mean ± SEM, with intergroup differences analyzed with one-way ANOVA used for statistical comparisons (* *p* < 0.05, ** *p* < 0.01, *** *p* < 0.001, and **** *p* < 0.0001).

Based on the clinical trials of JX-594, the function of IL-7, and the immune cell profiles of the TME after treatment with VVL-DD, we inserted the GM-CSF gene into the TK locus and the IL-7 gene into the A49 locus. This resulted in the construction of the recombinant virus VVL-GL7 (Figure 3A). Control viruses were also generated, including VVL-GF (expressing GM-CSF) and VVL-IL7 (expressing IL-7).

Quantification of GM-CSF and IL-7 secretion in cell culture supernatants was performed via ELISA (Figure 3B). Notably, the co-expression of both cytokines in VVL-GL7 did not alter their secretion profiles compared to viruses expressing either cytokine alone, as indicated by statistically equivalent ELISA measurements across groups.

TCID50-based titration of viral progeny at sequential timepoints revealed sustained replication capacity across DT6606, DT4994, and TB11381 pancreatic cancer models (Figure 3C). The engineered co-expression of GM-CSF and IL-7 correlated with enhanced viral replication efficiency in tumor cells. Notably, VVL-GL7 exhibited an increase in viral titers at the 72 h timepoint compared to parental viral controls, suggesting that cytokine co-expression potentiates viral fitness within the pancreatic tumor microenvironment.

The comparative analysis of cytotoxic activity demonstrated distinct profiles between VVL-DD and viral constructs engineered with GM-CSF and/or IL-7 (Figure 3D). The integration of these cytokines significantly modulated in vitro cytotoxic efficacy, as evidenced by reduced EC50 values in cytokine-armed viruses, which correlated with heightened tumor cell lethality. These data suggest a potential synergistic mechanism through which GM-CSF and IL-7 cooperatively enhance oncolytic activity.

### 3.4. Enhanced Anti-Tumor Efficacy of VVL-GL7 in Mouse Pancreatic Cancer Model

**Figure 4:**
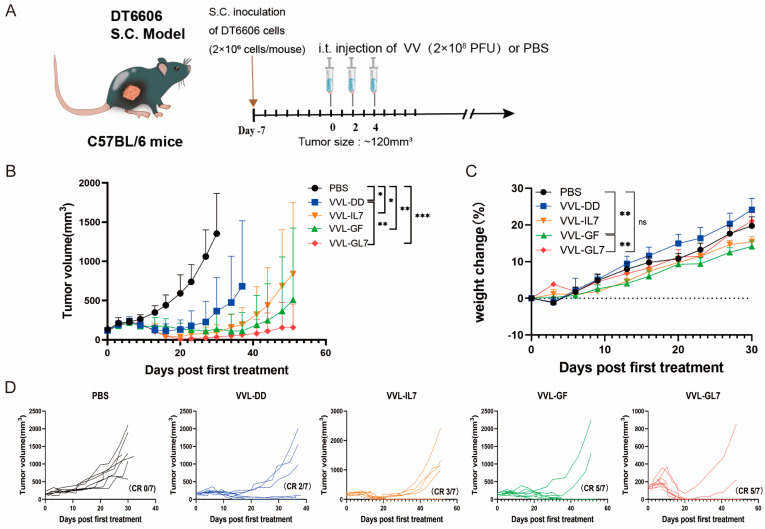
VVL-GL7 suppresses pancreatic tumor progression in vivo. (**A**) C57BL/6 mice bearing subcutaneous DT6606 tumors (~120 mm^3^) were randomized into five groups (n = 7): PBS, VVL-DD, VVL-GF, VVL-IL7, and VVL-GL7. Viruses (2 × 10^8^ PFU) or PBS were administered intratumorally every other day; altogether, 3 injections were performed; (**B**) tumor volume was measured every three days following treatment initiation. Data are presented as mean ± SD. Statistical analysis was performed using two-way ANOVA. * *p* < 0.05, ** *p* < 0.01, *** *p* < 0.001; (**C**) body weight changes. Data are presented as mean ± SEM. Statistical significance was calculated using two-way ANOVA. ** *p* < 0.01, ns: not significant; (**D**) tumor growth curves of individual animals. CR (complete response).

For the therapeutic assessment of the novel recombinant vaccine construct, a subcutaneous tumor model was established through DT6606 murine pancreatic carcinoma cell implantation. Mice with established tumors were treated with PBS (control), VVL-DD, VVL-GF, VVL-IL7, or VVL-GL7. Across the five treatment groups, three intratumoral injections of 2 × 10^8^ PFU vaccine virus were administered. Tumor growth was monitored, and survival rates were recorded (Figure 4A).

The results demonstrated that all viral treatments delayed tumor progression (Figure 4B). VVL-GL7 showed the strongest and most durable anti-tumor effect, with a higher percentage of mice achieving complete tumor clearance compared to viruses containing either GM-CSF or IL-7 alone (Figure 4D).

**Figure A1:**
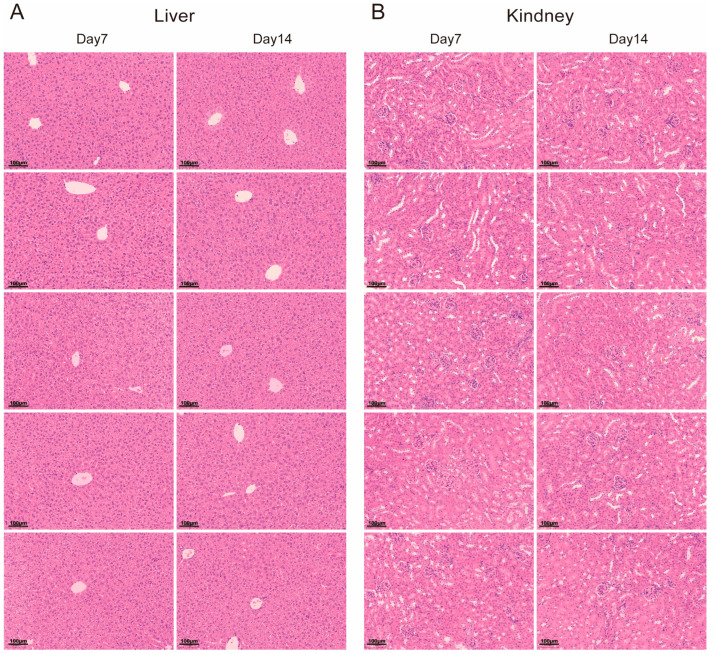
H&E staining of liver and kidney tissues at day 7 and day 14. (**A**) Liver and (**B**) kidney tissue sections stained with hematoxylin and eosin (H&E) to assess histological changes at two time points (day 7 and day 14).

**Figure A2:**
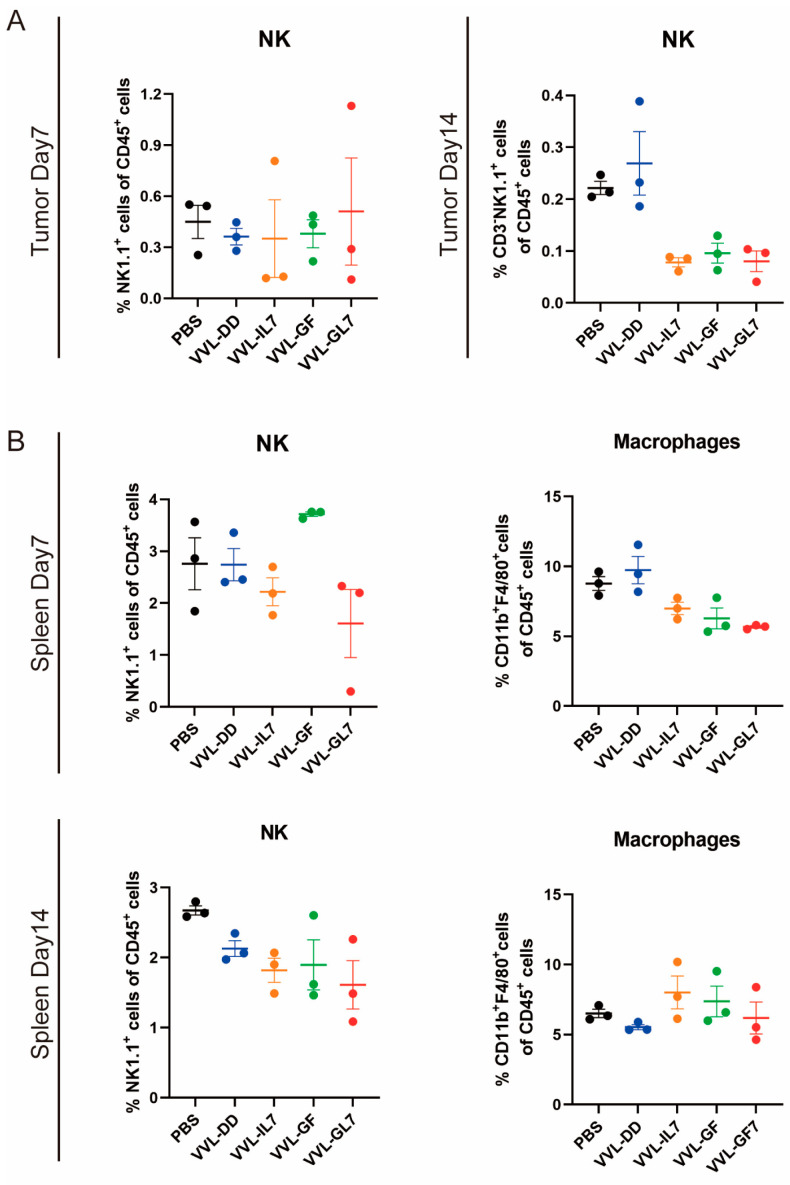
NK cell and macrophage proportions remain stable in tumor microenvironment and spleen. (**A**) Percentage of NK cells within CD45⁺ immune cells in tumors at Day 7 and Day 14 post-treatment. (**B**) Spleen NK cell and macrophage proportions within CD45⁺ cells at Day 14. Data are presented as mean ± SEM (n = 3). Statistical significance was determined using an unpaired Student’s *t*-test.

VVL-GL7 had minimal toxic side effects, with treated mice maintaining stable body weights compared to control groups throughout the treatment period (Figure 4C), or behavioral deficits (e.g., reduced activity, ruffled fur) in any treatment group. Additionally, H&E staining of liver and kidney tissues at 7- and 14-days post-treatment (Appendix A Figure A1) revealed no histopathological abnormalities (e.g., necrosis or inflammatory infiltrates) in VVL-GL7-treated mice compared to PBS controls, further underscoring the safety and tolerability of the treatment.

**Figure 5:**
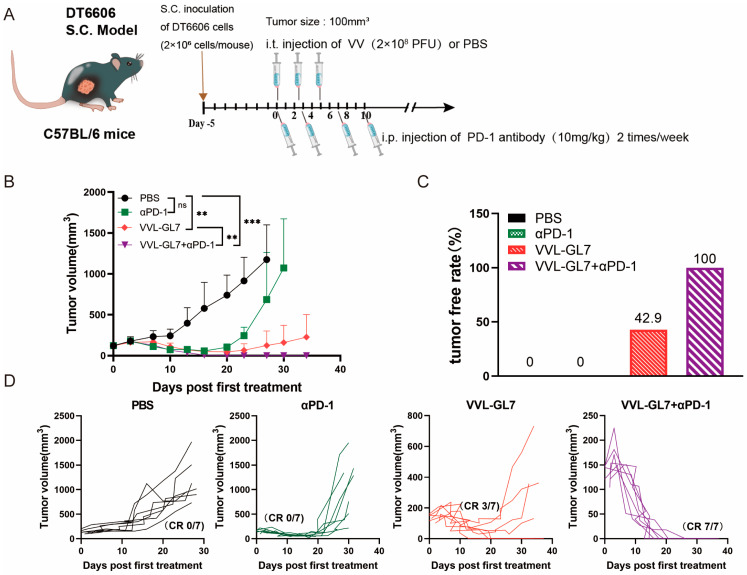
VVL-GL7 synergizes with PD-1 blockade to inhibit the growth of pancreatic cancer in mice. (**A**) Schematic diagram of the treatment protocol. C57BL/6 mice were s.c. inoculated with 2 × 10^6^ DT6606 pancreatic cancer cells. Once tumors reached approximately 100 mm^3^, mice were randomly divided into four groups (n = 7 per group): PBS control, αPD-1 alone, VVL-GL7 alone, and VVL-GL7 + αPD-1 combination. Virus (1 × 10^8^ PFU) was injected intratumorally every other day for a total of three doses, while αPD-1 antibody was administered intraperitoneally twice a week and continued throughout the study; (**B**) tumor volume was measured every three days after treatment began. Data are expressed as mean ± SD. Statistical significance was determined using two-way ANOVA. ** *p* < 0.01, *** *p* < 0.001, ns: not significant; (**C**) tumor-free rate. Percentage of mice with complete tumor regression in each group; (**D**) tumor growth curves of individual animals.

To further explore the potential of combining VVL-GL7 with immunotherapy, we tested its synergy with the PD-1 blockade. Mice were treated with a reduced viral dose of 1 × 10^8^ PFU combined with intraperitoneal injections of anti-PD-1 antibody (Figure 5A). Remarkably, the coadministration of VVL-GL7 with the PD-1 blockade completely eradicated tumors in all mice, with all achieving complete remission (Figure 5B–D). This was a significant improvement compared to either the virus or anti-PD-1 monotherapy alone, which only partially controlled tumor growth.

In summary, the combination of GM-CSF and IL-7 in VVL-DD significantly enhanced the immune response against tumors in mice. The addition of PD-1 blockades further boosted the therapeutic efficacy of VVL-GL7, offering a potent and safe treatment strategy for pancreatic cancer. The experimental data demonstrate that VVL-GL7 exhibits significant therapeutic efficacy against tumors, either as a standalone treatment or synergistically with PD-1 blockade, supporting its dual role in enhancing antitumor immunity.

### 3.5. VVL-GL7 Remodels Pancreatic Cancer Tumor Microenvironment in Mice

**Figure 6:**
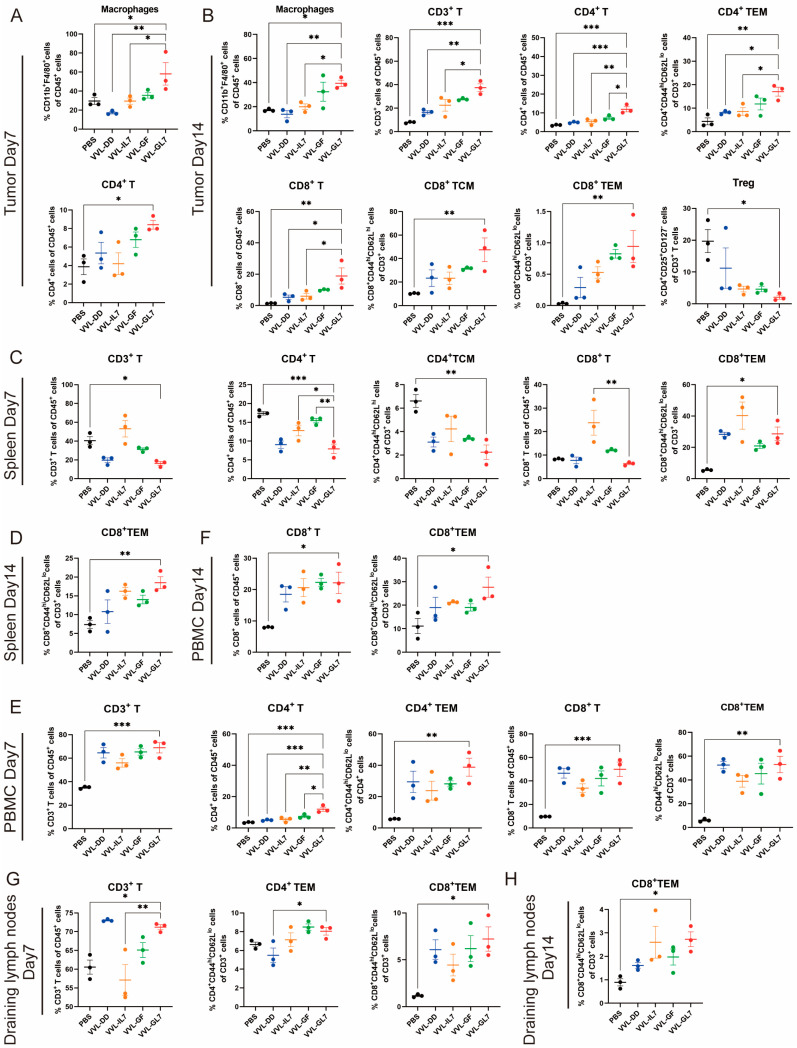
VVL-GL7 enhances systemic antitumor immunity. C57BL/6 mice that were subcutaneously inoculated with pancreatic cancer DT6606 cells were treated with VVL-GL7 and control viruses, and immune cells were detected in mouse tumor tissue (n = 3) (**A**,**B**), spleen (n = 3) (**C**,**D**), peripheral blood (n = 3) (**E**,**F**), and draining lymph nodes (n = 3) (**G**,**H**) using flow cytometry on days 7 and 14 after initial treatment. Data were expressed as mean ± SEM. Statistical significance was determined using an unpaired Student’s *t*-test. * *p* < 0.05, ** *p* < 0.01, *** *p* < 0.001.

To evaluate the immune-modulatory effects of VVL-GL7, immune cell populations were analyzed in tumor tissues, spleen, peripheral blood, and draining lymph nodes on days 7 and 14 post-treatment using flow cytometry (Figure 6). In the tumor microenvironment, VVL-GL7 significantly increased macrophage infiltration on day 7 compared to other groups, indicating enhanced innate immune activation. By day 14, VVL-GL7 treated tumors exhibited a marked increase in CD3^+^ T-cells, including both CD4^+^ and CD8^+^ effector memory T (TEM) cells. Furthermore, regulatory T-cells (Tregs) were moderately reduced in the VVL-GL7 group (Figure 6A,B).

Additionally, this amplified systemic immune responses in the spleen, peripheral blood, and draining lymph nodes. Flow cytometry analysis showed that VVL-GL7 treatment reduced CD3^+^ and CD4^+^ T-cells in the spleen compared to the PBS group. Despite this, the CD8^+^ TEM cells were significantly increased on day 7 and remained higher than all other groups on day 14. These findings suggest that VVL-GL7 selectively enhances cytotoxic TEM cells, potentially compensating for the overall reduction in CD3^+^ and CD4^+^ T-cells (Figure 6C–H).

Flow cytometric measurements of peripheral blood specimens revealed a marked elevation in CD8^+^ T lymphocyte populations (including TEM subsets) within the VVL-GL7 cohort, with statistically significant differences observed at both 7-day and 14-day monitoring intervals compared to control groups. This systemic immune activation was further supported by the increased frequency of CD3^+^ and CD8^+^ T-cells in the draining lymph nodes, particularly the TEM subset, in VVL-GL7-treated mice compared to other groups (Figure 6E–H).

**Figure 7:**
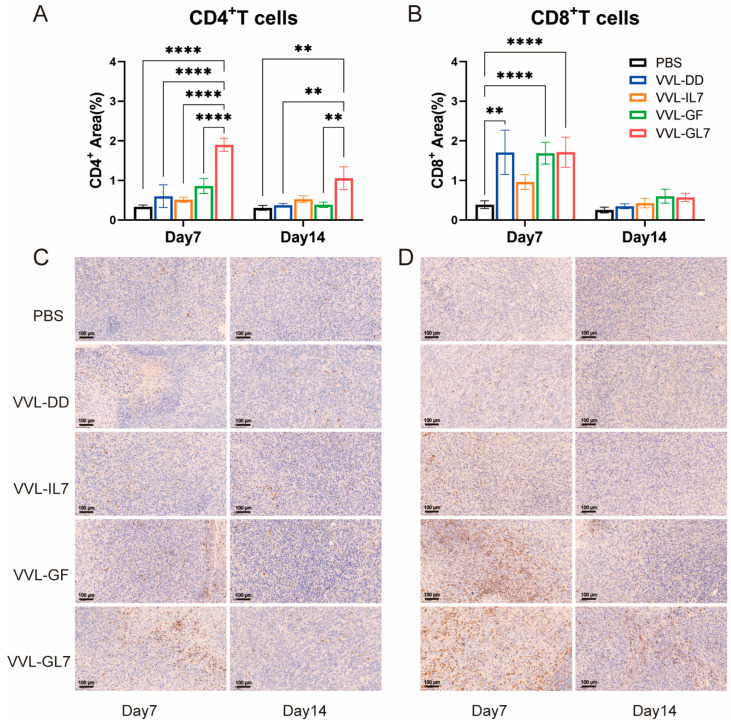
Immunohistochemical analysis and quantification of CD4^+^ T-cells and CD8^+^ T-cells in the TME. (**A**) Quantification of CD4^+^ T-cells in the tumor microenvironment on days 7 and 14 after treatment with PBS, VVL-DD, VVL-IL7, VVL-GF, and VVL-GL7. Five microscopic fields per tumor section (×20). n = 3; (**B**) CD8^+^ T-cell density was calculated in five microscopic fields per tumor section (×20). n = 3, The data in (**A**,**B**) were expressed as mean ± SEM. Statistical significance was determined using an unpaired Student’s *t*-test. ** *p* < 0.01, **** *p* < 0.0001; (**C**) representative immunohistochemical images of CD4^+^ T-cells on days 7 and 14 for each treatment group. Scale bars: 100 μm; (**D**) representative immunohistochemical images of CD8^+^ T-cells at both time points. Scale bars: 100 μm.

Tumor-infiltrating CD4^+^ and CD8^+^ T lymphocytes were quantified via immunohistochemistry. VVL-GL7 treatment induced a significant increase in CD4^+^ T-cell density relative to controls at both 7- and 14-day time points (Figure 7A,B). In contrast, VVL-GF administration elevated CD8^+^ T-cell frequencies significantly compared to PBS at day 7 (Figure 7C,D).

### 3.6. VVL-GL7 Cures the Peritoneally Disseminated Pancreatic Cancer in Syrian Hamster

Other groups have reported that Syrian hamsters were sensitive to human GM-CSF [40] and IL-7, while our group reported that human IL-12, IL-21, and IL-2 had a function in Syrian hamsters [38,41]. These results mean that Syrian hamsters have similar immune systems to humans. So, we constructed a human version of VVL-hGL7 and tested it in human and Syrian hamster pancreatic cancer models.

**Figure 8:**
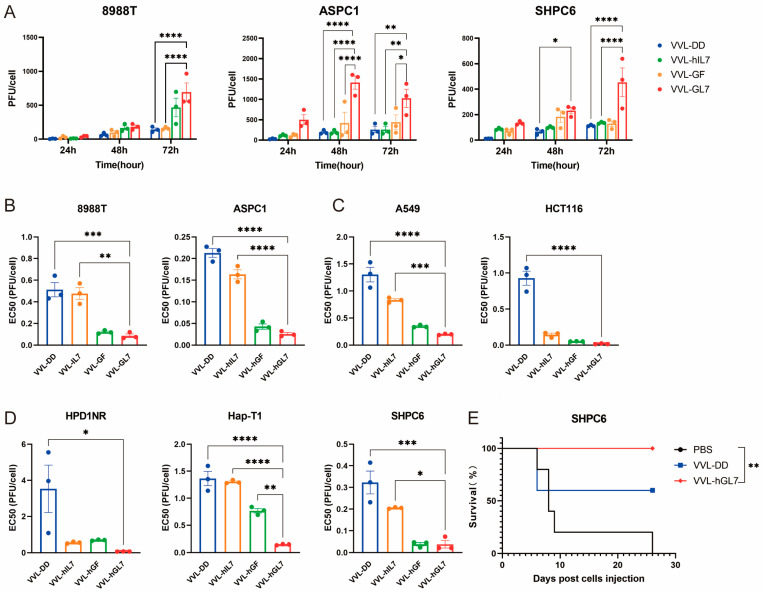
VVL-hGL7 prolongs survival of Syrian hamsters with peritoneally disseminated pancreatic cancer. (**A**) Replication of VVL-hGL7 and control viruses was measured by TCID50 assay in human and hamster pancreatic cancer cells at 24 h, 48 h, and 72 h post-infection (n = 3); (**B**) cytotoxic effects of VVL-hGL7 and control viruses in human pancreatic cancer cell lines (ASPC1 and 8988T) (n = 3); (**C**) the EC50 values were subsequently compared between the VVL-hGL7 and control viruses, using the MTS assay in HapT-1 and HPD1NR cells (n = 3); (**D**) comparative analysis of VVL-hGL7 versus control vectors in human non-small cell lung cancer (A549) and colorectal cancer (HCT116) cellular systems. Statistical determination via one-way ANOVA with mean ± SEM (* *p* < 0.05, ** *p* < 0.01, *** *p* < 0.001, **** *p* < 0.0001); (**E**) SHPC6 tumors were established intraperitoneally in Syrian hamsters. Hamsters were treated intraperitoneally with PBS, 2 × 10^7^ PFU/mL VVL-DD, or VVL-hGL7 on days 4, 6, and 8 post-tumor implantation. Statistical significance was calculated using Kaplan–Meier survival analysis with Log-rank (Mantel-Cox) test.

VVL-hGL7 exhibited superior replication capacity compared to the control viruses in human and Syrian hamster pancreatic cancer cells, reinforcing its potential as a therapeutic agent (Figure 8A).

All engineered viral constructs exhibited potent cytotoxic activity, as evidenced by low EC50 values across diverse tumor models (human pancreatic, lung, colorectal cancer, and Syrian hamster pancreatic carcinomas), suggesting broad therapeutic applicability (Figure 8B–D). Notably, VVL-hGL7 demonstrated significantly enhanced oncolytic efficacy compared to VVL-DD, directly linking GM-CSF/IL-7 co-expression to improved cytotoxic potency in vitro (Figure 8B–D).

We established a peritoneally disseminated pancreatic cancer model via intraperitoneal injection of SHPC6 using Syrian hamsters. Intraperitoneal VVL-hGL7 administration conferred a significant survival advantage over VVL-DD treatment, as evidenced by longitudinal monitoring (Figure 8E).

## 4. Discussion

To overcome the limited therapeutic efficacy of VV, the viral backbone and therapeutic gene need to be optimized. We found that VVL-DD exhibited strong antitumor capacity in pancreatic cancer models with Kras or/and p53 mutation by enhancing the accumulation of macrophages and T-cells, which provides a basis for further construction of therapeutic viruses based on this backbone. The efficacy of VVL-DD was closely related to its ability to mobilize macrophages and T-cells. Macrophage is a kind of key immune cell in the tumor microenvironment that plays an important role in promoting antigen presentation and modulating inflammatory responses [42,43,44,45]. Our results suggest that VVL-DD significantly increased the number of macrophages within the tumor, and this is important for improving the killing effect of this viral backbone on the tumor.

Combining the data of viral replication and cytotoxicity, we found that the increased cancer cell-killing ability of VVL-GL7 was not solely due to enhanced viral replication. Although higher viral titers can contribute to oncolysis, the expression of GM-CSF and IL-7 may have additionally influenced the tumor cells’ susceptibility to viral killing. The data indicate that the relationship between viral replication and cytotoxicity may be affected by the immunomodulatory effects of the expressed cytokines, rather than a direct linear correlation.

VVL-GL7 demonstrated significantly enhanced anti-tumor effects, not only by inducing potent T-cell responses locally in the tumor [46] but also by enhancing memory T-cell production on a systemic scale [46,47]. Despite the demonstrated potential of viruses combining immunizing factors like VVL-GL21 co-expression GM-CSF/IL-21 [20], the absence of T-cell infiltration and the defective memory response in pancreatic cancer still pose an unsolved issue. In this study, we engineered VVL-GL7 designed to augment T-cell survival and memory differentiation rather than transient cytotoxicity. IL-7 significantly increases the content of memory T-cells and maintains their long-term survival by activating the STAT5-Bcl2 pathway [34,35]. While IL-21 temporarily enlarges memory T-cells, its reliance on STAT3 to enhance short-term effector function might result in terminal differentiation and exhaustion [48]. The design concept of VVL-GL7 focuses on long-lasting immune memory rather than purely enhancing cytotoxicity, which provides a new opportunity to treat immune ‘cold’ tumors. This phenomenon was particularly pronounced in tumor tissues, especially in CD4^+^ and CD8^+^ T-cell subsets [47]. GM-CSF enhances the activation of immune responses in the tumor microenvironment by promoting the mobilization and maturation of antigen-presenting cells (APCs) [46,49]. We found that VVL-GL7 was able to mobilize more macrophages after the treatments.

On the other hand, IL-7 mainly promotes T-cell survival and proliferation [37], especially in CD4^+^ memory T-cells and CD8^+^ memory T-cell subsets [36]. Our study demonstrated that VVL-GL7 led to a substantial augmentation in the number of memory T-cells within tumor tissues, particularly on day 14, and the proportion of CD4^+^ and CD8^+^ TCM and TEM cells was significantly higher than that of controls. While tumor-rechallenge experiments are needed to fully confirm long-term immunity, the observed expansion of memory T-cell subsets (CD44^+^ CD62L^−^TEM) and sustained tumor-free survival strongly suggest that VVL-GL7 induces durable immune memory. Future studies will include rechallenge experiments in congenic models and single-cell sequencing to track clonal T-cell persistence [50,51]. This suggests that the synergistic effect of GM-CSF and IL-7 can simultaneously enhance the mobilization of immune cells and long-term accumulation of memory T-cells in the TME, resulting in a sustained anti-tumor effect [52,53].

This study revealed the important role of myeloid cells in vaccine virus therapy [54]. VVL-GL7 significantly increased the number of macrophages in the tumor microenvironment on day 7, and this mobilizing effect persisted on day 14 [55]. GM-CSF is a well-known myeloid regulator, which enhances the antigen-presenting function of macrophages and exerts a direct antitumor effect in the tumor microenvironment [44,46]. Macrophage aggregation not only enhances the presentation of tumor antigens but may also enhance direct attack on tumor cells by releasing pro-inflammatory factors [56].

Our study focused on demonstrating the significant effect of VVL-GL7 in T-cell activation and memory T-cell accumulation [57]. The initial tumor analysis on day 7 revealed modest CD3^+^ T lymphocyte infiltration without statistical significance. By day 14, VVL-GL7 intervention induced robust expansion of central/effector memory T-cell reservoirs (CD4^+^/CD8^+^ TCM/TEM subsets), with CD8^+^ TEM populations showing pronounced spatial accumulation in both tumor tissue and the spleen, providing evidence of persistent immune memory within the tumor microenvironment [58,59].

Memory T-cells play an important role in anti-tumor immunity as they are able to respond rapidly and proliferate upon re-encountering tumor antigens [58,60]. We hypothesize that IL-7 insertion enhances the survival of memory T-cells [61], while GM-CSF collectively enhances the immune memory effect of T-cells by increasing antigen presentation efficiency and stimulating T-cell activation [46]. GM-CSF and IL-7 work together to create a coordinated immune cascade. IL-7 protects the survival of tumor-reactive memory T-cell clones, while GM-CSF recruits and licenses myeloid sentinels to break down immunosuppressive barriers. A self-sustaining loop of tumor eradication and immune memory reinforcement is the result of this combination. This may explain the sustained antitumor effect we observed on day 14, which was more pronounced in combination with PD-1 inhibitors, leading to complete tumor regression in mice [62].

The ultimate confirmation of this research was achieved using the pancreatic cancer model in Syrian hamsters, which has emerged as the gold standard for preclinical investigations due to its distinctive pathological features. Additionally, in the disseminated Syrian hamster intraperitoneal model, tumor progression is similar to end-stage human PDAC [63]. We successfully constructed a human vaccinia virus containing human GM-CSF and IL-7 and validated its therapeutic effect in the hamster pancreatic cancer model. The experimental results showed that VVL-hGL7 was able to significantly inhibit tumor growth, which lays the foundation for its future clinical application. Future studies should further explore the safety and adaptability of this virus and its combined effect with other immunotherapies, with the aim of providing more effective treatments for cancer patients.

The therapeutic design of VVL-GL7 integrates key translational features informed by prior clinical successes and limitations of oncolytic viruses. Dosing strategies were adapted from phase I trials of JX-594 [NCT01171651] [64], with our preclinical regimen demonstrating efficacy without toxicity in immunocompetent models (Figure 4C). Notably, VVL-GL7 achieved tumor control in the abdominal dissemination model of pancreatic cancer in Syrian hamsters (Figure 8E), mirroring the systemic administration potential of vaccinia virus-based therapies [38].

Safety-wise, VVL-GL7’s tumor-selective replication (Figure 3C) aligns with the established safety profile of oncolytic vaccinia viruses in humans [12]. However, two translational hurdles warrant attention: Pre-existing anti-vaccinia immunity may attenuate viral spread. Transient immunosuppression (e.g., low-dose cyclophosphamide) has proven effective in clinical trials to enhance oncolytic virus potency [38]. A fibrotic pancreatic TME could limit viral penetration. Co-administration with stroma-modulating agents (e.g., PEGylated hyaluronidase) may synergize with VVL-GL7, as shown preclinically for other oncolytic platforms [65].

Critically, VVL-GL7’s IL-7 drove memory T-cell persistence (Figure 5C,D). By sustaining tumor-specific memory T-cell pools, VVL-GL7 may reduce the dosing frequency and long-term immune attrition—an advantage for managing pancreatic cancer’s immuno-suppressive plasticity.

Collectively, the data position VVL-GL7 as a potent immunotherapeutic candidate, driving both immune cell recruitment and durable tumor-specific memory responses within the TME. These observations not only underscore the utility of this novel virus but also suggest its potential for broader application in cancer immunotherapy.

## Data Availability

The datasets used and/or analyzed in the current study were all included in this manuscript. If there is a further need, it can be provided upon reasonable request to the corresponding author.

## Abbreviations

The following abbreviations are used in this manuscript:

APCs	Antigen-presenting cells
CR	Complete response
CTLA-4	Cytotoxic T-lymphocyte antigen 4
DC	Dendritic cells
DMEM	Dulbecco’s Modified Eagle Medium
EC50	Half maximal effective concentration
GM-CSF	Granulocyte-Macrophage Colony-Stimulating Factor
ICI	Immune checkpoint inhibitor
IL-7	Interleukin-7
MOI	Multiplicity of Infection
OV	Oncolytic virus
PBS	Phosphate-Buffered Saline
PD-1	Programmed cell death protein 1
TCM	T central memory cells
TEM	T effector memory cells
TME	Tumor microenvironment
Tregs	Regulatory T cells
VV	Vaccinia virus
