# Correction: Yan et al. Oncolytic Vaccinia Virus Armed with GM-CSF and IL-7 Enhances Antitumor Immunity in Pancreatic Cancer. *Biomedicines* 2025, *13*, 882

**DOI:** 10.3390/biomedicines13082011

**Published:** 2025-08-19

**Authors:** Wenyi Yan, Yujing Xuan, Ruimin Wang, Ziyan Huan, Yu Guo, Huilin Dun, Lihua Xu, Ruxia Han, Xianlei Sun, Lingling Si, Nicholas Robert Lemoine, Yaohe Wang, Pengju Wang

**Affiliations:** 1 Sino-British Research Centre for Molecular Oncology, National Centre for International Research in Cell and Gene Therapy, State Key Laboratory of Esophageal Cancer Prevention & Treatment, School of Basic Medical Sciences, Academy of Medical Sciences, Zhengzhou University, Zhengzhou 450001, China; ywy201377@126.com (W.Y.); nick.lemoine@nihr.ac.uk (N.R.L.);; 2 Department of Pathology, Zhengzhou People’s Hospital, Fifth Clinical Medical College of Henan University of Chinese Medicine, Zhengzhou 450003, China; 3 Centre for Biomarkers & Biotherapeutics, Barts Cancer Institute, Queen Mary University of London, London EC1M 6BQ, UK

## Error in Figure

In the original publication [1], there was a mistake in Figure 2A as published. Specifically, three image panels (CD8 and F4/80 staining on Day 10) were inadvertently duplicated during the figure rearrangement process. The corrected Figure 2 appears below. The authors state that the scientific conclusions are unaffected. This correction was approved by the Academic Editor. The original publication has also been updated.

## Figures and Tables

**Figure 2:**
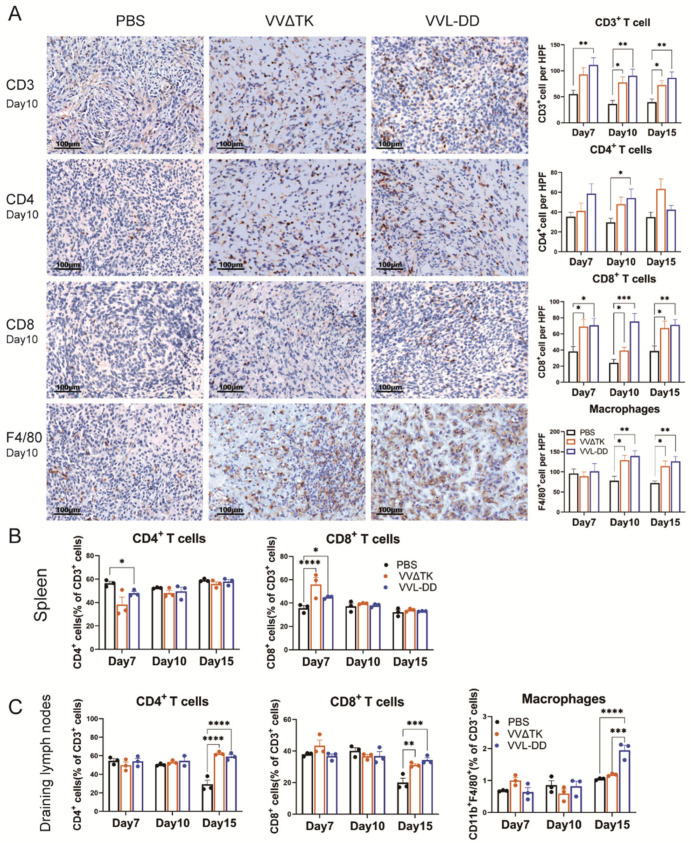
Immune cell profiles of tumor tissue, spleen, and draining lymph nodes of mice after treatments. (**A**) Quantification of immune cell populations in tumor tissues over time post-treatment. Immunohistochemistry (IHC) assay was used to assess the presence of CD3^+^ T-cells, CD4^+^ T-cells, CD8^+^ T-cells, and macrophages (F4/80^+^) on days 7, 10, and 15 post-treatments with PBS, VVΔTK, or VVL-DD. Representative images of IHC staining for CD3^+^ T-cells, CD4^+^ T-cells, CD8^+^ T-cells, and macrophages on day 10. Data are presented as mean ± SEM. * *p* < 0.05, ** *p* < 0.01, *** *p* < 0.001. (**B**,**C**) Spleen and lymph node specimens harvested on post-treatment days 7/10/15; flow cytometric analysis of tissue-derived single-cell suspensions for lymphocyte profiling (n = 3). Data represent mean ± SEM, with two-way ANOVA used for statistical comparisons (* *p* < 0.05, ** *p* < 0.01, *** *p* < 0.001, **** *p* < 0.0001).
